# Effect of pH of In-Office Bleaching Gels and Timing of Fluoride Gel Application on Microhardness and Surface Morphology of Enamel

**DOI:** 10.1155/2023/1041889

**Published:** 2023-09-05

**Authors:** Fariba Motevasselian, Hamid Kermanshah, Dorara Dortaj, Frank Lippert

**Affiliations:** ^1^Department of Restorative Dentistry, School of Dentistry, Tehran University of Medical Sciences, Tehran, Iran; ^2^Department of Cariology, Operative Dentistry and Dental Public Health, Oral Health Research Institute, Indiana University School of Dentistry, Indianapolis, IN, USA

## Abstract

**Objective:**

To assess microhardness (VH) of enamel treated with two in-office bleaching agents with different pH and to study the effect of post- and prebleaching fluoride therapy.

**Materials and Methods:**

Eighty bovine incisors were divided into eight groups: G1-Unbleached group; G2-2% NaF; G3-Pola Office (pH = 3.8); G4-Pola Office+ (pH = 7); G5-Pola Office followed by 2% NaF; G6-2% NaF followed by Pola Office; G7-Pola Office+ followed by 2% NaF; G8-2% NaF followed by Pola Office+. Bleaching was conducted 3x with 1-week intervals (T1/T7/T14). Specimens were kept in artificial saliva. VH was measured at T1, T7, and T14. Data were analyzed using repeated measure ANOVA. *S*urface morphology was assessed using scanning electron microscopy.

**Result:**

There was no significant difference among the groups at T1. No significant difference was found between G3 and G4 at all intervals. 2% NaF (G5/G6 vs. G3) significantly prevented softening at T7 and T14. Some nonsignificant hardening was observed for 2% NaF for G7/G8 vs. G4. At T14, G3 showed the lowest VH values. G5 showed higher VH values compared to other groups apart from G6–G7. No relationship between bleaching protocols and surface morphology was observed.

**Conclusion:**

Pola Office caused the most softening. 2% NaF gel application after Pola Office bleaching was effective in recovering enamel hardness. Fluoride application after Pola Office+ bleaching provided little benefit.

## 1. Introduction

Effects of bleaching agents on the enamel surface have been a clinical concern as they can lead to enamel demineralization, hardness, and surface morphological changes [1, 2].

In-office bleaching utilizes high levels of hydrogen peroxide (HP) and is suitable when patients desire to obtain faster bleaching results compared to at-home bleaching [3].

There is a safety concern in using high concentrations of peroxides. Some studies have reported structural alterations of the enamel, characterized by increasing roughness and loss of mineral structure as a result of exposing dental hard tissues to high concentrations of bleaching agents [1, 4, 5].

Bleaching agents have different pH values ranging from highly acidic to highly basic. Acidic bleaching agents may help to keep hydrogen peroxide stable and improve stability and lifetime of the product [6]. However, the acidic pH of the bleaching product may cause deleterious effects on the tooth structure. In contrast, products with neutral or alkaline pH produce greater bleaching efficacy under in vitro conditions, due to constant hydrogen peroxide dissociation. In addition, these bleaching agents do not produce adverse effects on the enamel surface [7, 8]. However, it is not clear yet whether enamel surface alteration is caused by the low pH or HP per se [8–10].

The importance of understanding the effect of pH of bleaching agents on the tooth enamel is important to help determining the potential adverse effects [11]. In this context, the microhardness test has been used to assess whether demineralization occurs [8].

Some strategies have been proposed to prevent changes in the enamel surface, such as topical fluoride application. Historically, fluoride has been the first attempt in dental practice used for preventive purposes [12]. Toothpastes, mouth rinses, and topical gels have been developed over years showing significant results. However, there is some controversy as previous studies regarding the effect of bleaching agents and fluoride products on tooth enamel microhardness yielded conflicting results [2]. This may be due to the type, concentration, pH, and the formulation of bleaching agent used [11]. Thus, we aimed to evaluate the efficacy of two commercial in-office bleaching gels with different pH values from the same company to reduce the variation of formulation of bleaching agents as a cofounding factor. The primary goal of conducting this in vitro research was to evaluate the effect of pH of the bleaching agent and topical fluoride application before or after bleaching on microhardness loss of enamel. scanning electron microscopy (SEM) images of enamel surfaces were also assessed. Our null hypotheses were that: (1) the pH value of bleaching agents has no effect on the microhardness of enamel and (2) topical fluoride application has no beneficial effect on enamel microhardness.

## 2. Materials and Methods

### 2.1. Ethical Considerations

For research studies using human and animal subjects, the research proposals including the method parts should be submitted to the research ethics committee of Tehran University of Medical Sciences. The committee should approve whether the method conform to the ethical guidelines (such as World Medical Association (WMA) Declaration of Helsinki) prior to the research practice.

### 2.2. Specimen Preparation

Bovine permanent incisor teeth were obtained from an abattoir. The cattle were younger than 5 year olds. The teeth were stored in freshly prepared 1% chloramine-T trihydrate solution up to 1 week and thereafter in distilled water at 4°C [13]. All teeth were examined under magnification (×20) to detect enamel cracks or fractures and other defects. The labial enamel surface of the bovine incisors was subjected to wet grinding with 800, 1,000, and 2,000 grit silicon carbide papers to obtain flat enamel surfaces. The samples were then rinsed under running deionized water to remove debris.

One specimen (5 × 5 mm) was obtained from the middle third of the buccal surface using water-cooled separating discs. The specimens were horizontally embedded in a plastic mold by self-curing acrylic resin, leaving their labial surfaces uncovered. The specimens were randomly distributed into eight experimental groups (*n* = 10 per group).

### 2.3. Test Products

Table 1 provides details about the experimental groups and commercially available products that were employed in the present study. The composition of the two in-office bleaching agents is shown in Table 2.

The pH values of the bleaching agents and topical fluoride gel were measured using a portable pH meter (PT-15, Sartorius AG, Gottingen, Germany) with a direct electrode, which was calibrated with standard buffer solutions at pH 4.0 and 7.0 prior to analysis.

### 2.4. Treatment Procedure

Throughout the entire experiment, the specimens were kept in artificial saliva (pH 7.0) at room temperature. The composition of this solution was 1.5 mmol/L Ca, 0.9 mmol/l PO_4_, 150 mmol/L KCl, and 0.1 mol/L Tris buffer [18]. The artificial saliva was changed daily.

For the bleached groups, the specimens received a HP gel application according to the manufacturer's instructions. The bleaching agent was applied evenly on the enamel surface of the specimens in a 2 mm-thick layer using a microbrush and remained in contact with the enamel surface until it was removed with gauze, totaling three bleaching gel applications at each session. After the third application, the bleaching gel was removed with gauze. Then, the specimens were rinsed with tap water and dried with an air syringe. Two percent neutral sodium fluoride was applied to the specimens according to Table 1 with a microbrush and remained in contact for 4 min until it was removed with gauze. This procedure was repeated after 7 (T7) and 14 (T14) days, resulting in three bleaching sessions. The specimens were then washed with running water and dried with mild air blow.

### 2.5. Microhardness Assessment

Vickers surface microhardness (VH) was measured at base line (T0) and after every bleaching application (T1, T7, and T14) with a microhardness tester (V-Test II, Bareiss, Germany). Three indentations at a load of 100 g and a dwelling time of 10 s were performed on the surface of each specimen, with a distance of 50 *µ*m between them [10, 19]. The mean VH was calculated. After completion of the VH assessment, the specimens were stored in artificial saliva at room temperature for a period of 24 hr, until they were analyzed by SEM.

### 2.6. Scanning Electron Microscopy

Two specimens from each group were randomly selected, dried and fixed on aluminum stubs and then sputter-coated with gold–palladium. The surface morphology of enamel was examined using a scanning electron microscope (TESCAN BRNO-Mira3, Brno, Czech Republic) at 2,500x magnifications by three evaluators.

### 2.7. Statistical Analysis

The minimum sample size was calculated to be 10 for each group, based on a previous study by Kutuk et al. [20], considering *α* = 0.05, *β* = 0.2, standard deviation = 58, and effect size = 0.46 using one-way ANOVA power analysis to detect significant difference for 60 units (PASS11, Chicago, IL). The means of hardness of five groups were extracted from Kutuk et al. [20]. The means were 420, 415, 425, 473, and 475.

The statistical analysis of data was performed using SPSS version 25 (SPSS Inc., Chicago, IL, USA). All statistical analyses were carried out at a significance level of 0.05. Mean values of VH of samples in the experiment were expressed as means ± standard deviation. Repeated measures ANOVA was used to analyze the effects of the two main factors, pH and fluoridation, on VH of enamel. The normality of data in each time and each study group were evaluated using Shapiro–Wilk test (*p* > 0.05). Then, the variations for VH values in time were analyzed separately in each group. Pairwise comparison of groups was performed using post hoc Tamhane due to the heterogeneity of the variances. The type 1 error in this research was considered to be 0.05.

## 3. Results

The pH values for Pola Office 35%, Pola Office + 37.5%, and fluoride gel were 3.85, 7.08, and 7.20, respectively.

**Figure 1:**
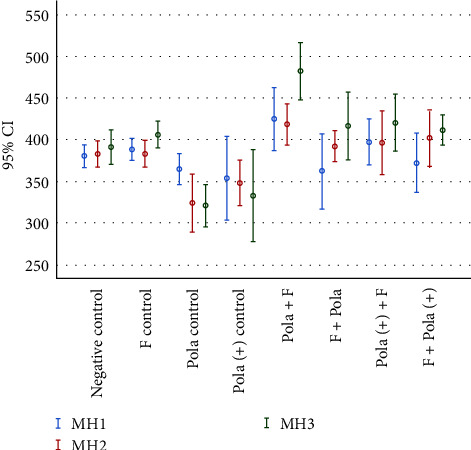
Mean and standard deviation of enamel microhardness vs. time after bleaching treatment and topical fluoride application (*n* = 10). Abbreviation: enamel microhardness after first, second, and third session of treatment (MH1, MH2, and MH3).

Sound enamel (baseline) hardness values (T0) were (380.38 ± 20.58) for all groups with no statistically significant differences between groups (*p*=0.98). The ANOVA revealed the effect of frequency of bleaching time and topical fluoride gel with differences between experimental groups (*p* < 0.05). The VH data and results of the statistical analysis are presented in Figure 1 and Table 3. There was no significant difference between the experimental groups and control groups at the T1 of the experiment (*p* > 0.05). Average VH values at T7 did not significantly differ between the experimental groups and control groups. Furthermore, no significant difference was found between Pola Office and Pola Office+ (*p*=0.999). However, topical fluoride application either before or after bleaching with Pola Office increased VH values compared to the Pola Office bleaching group (*p*=0.043). There was no significant difference between the two former groups (*p*=0.877). Pola Office and the fluoridation group showed higher VH compared to Pola Office+ (*p*=0.013).

At T14, negative and positive control groups showed significantly higher hardness values compared to the Pola Office group (*p*=0.004 and *p* < 0.001, respectively).

Topical fluoridation both before and after bleaching with Pola Office at T14 showed higher hardness compared to the Pola Office group (*p*=0.01 and *p* < 0.001, respectively). However, no difference was found between fluoridation before vs. after groups (*p*=0.284). VH values for Pola Office+ and fluoridation before or after bleaching treatment were higher than the Pola Office group (*p* < 0.001 and *p*=0.002, respectively). However, there was no significant difference between fluoridation before vs. after groups (*p*=0.999).

In addition, topical fluoride application after bleaching with Pola Office showed higher VH values compared to the positive control group (*p*=0.015).

Pola Office bleaching and fluoridation group showed higher hardness compared to the Pola Office+ group (*p*=0.003). The former group also revealed increased hardness compared to the fluoridation and Pola office+ bleaching group (*p*=0.029).

Different trends were found for each group when comparing among T1, T7, and T14. No significant change in enamel hardness was observed for the positive and negative control groups throughout the experimental period (*p*=0.094 and *p*=0.655, respectively). Pola Office caused a significant reduction in VH at T14 compared to T1 (*p*=0.008). No difference in VH was detected at different interval times for the Pola Office+ group (*p*=0.634). Significant increases in VH were observed at T14 compared to T7 following bleaching with Pola Office and topical fluoridation (*p*=0.005). However, topical fluoride application before treatment sessions with Pola Office had no significant effect on VH at different interval times (*p*=0.107). Similar scenarios were found for Pola Office+ and fluoridation protocols either before or after bleaching (*p*=0.094 and *p*=0.418, respectively).

**Figure 2:**
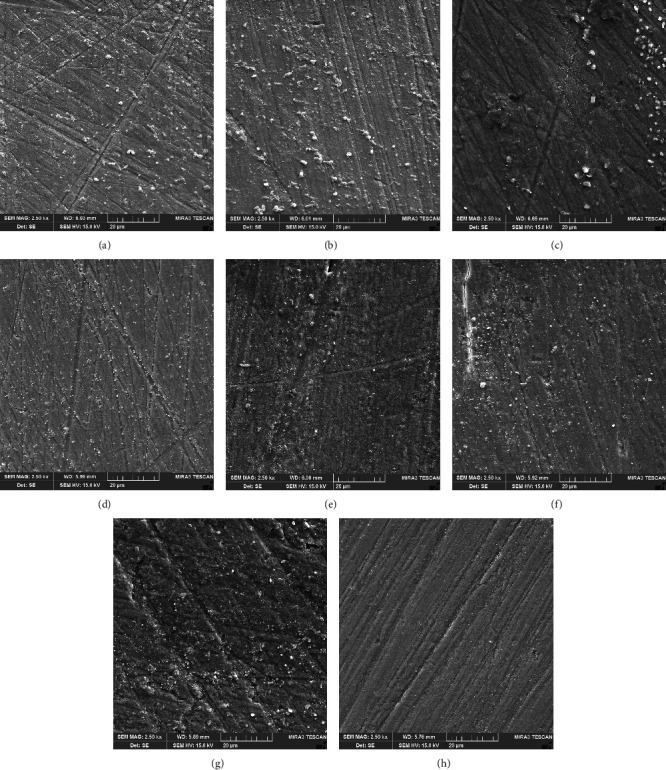
Scanning electron micrograph (SEM) of enamel specimens after third session of treatment (14^th^ day). (a) Negative control specimen (unbleached). (b) Positive control specimen (2% neutral sodium fluoride) (2% NaF). (c) Specimen bleached with Pola Office. (d) Specimen bleached with Pola Office+. (e) Specimen bleached with Pola Office and treated with 2% NaF. (f) Specimen treated with 2% NaF 24 hr before bleaching process with Pola Office. (g) Specimen bleached with Pola Office+ and treated with 2% NaF. (h) Specimen treated with 2% NaF 24 hr before bleaching process with Pola Office+ (×2,500).

SEM images are shown in Figure 2(a)–2(h). It can be seen that SEM images did not show considerable differences among specimens treated with the bleaching agents of different pH values. The only exception was for rounding of sharp edges of random scratch lines in the Pola Office group, which were the result of the specimen preparation procedure (Figure 2(c)). The images barely showed porosities or irregularities on the enamel surface following bleaching with different bleaching agents (Figures 2(c) and 2(d)). SEM images showed similar morphologies in the groups treated with NaF. A superficial coating was observed in these groups, which was likely a CaF_2_-containing mineral deposit (Figures 2(b) and 2(e)–2(h)).

## 4. Discussion

The first null hypothesis, that pH values of bleaching agents do not affect the microhardness of enamel, was rejected. The results ranged from no effects to significant decrease in hardness of enamel. The second null hypothesis, that topical fluoride application has no beneficial effect on enamel microhardness, was also rejected. Groups treated with fluoride showed increased enamel microhardness compared to control and experimental groups.

In-office bleaching is usually performed in two to three sessions with a 1-week interval to achieve optimum esthetic results, since a single in-office bleaching session seems not to be sufficient to whiten teeth effectively and satisfy the patient's expectations [21]. However, several studies have reported a decrease in enamel microhardness after bleaching, especially after in-office bleaching using high concentrations of HP [1, 4] and product with different pH values [3, 11]. It has been reported that fluoridation may increase enamel resistance to demineralization during bleaching treatment. However, there is no general agreement on the optimum time for fluoridation. On one hand, it has been recommended that the application of fluoride gel prior to the bleaching treatment might increase the demineralization resistance of enamel. On the other hand, it has been suggested that fluoridation after bleaching treatment might be more effective in improving enamel resistance [2, 22, 23].

Concerns regarding bleaching agents have mainly centered around lower pH based materials [10, 24, 25]. The Pola Office+ formulation had a pH of 7.08, which was not expected to have any detrimental effects on enamel as its pH was above the critical pH for enamel dissolution [26]. However, Pola Office had a pH of 3.85 which may likely explain the present findings (Figure 1).

Controversial findings have been reported for enamel microhardness changes as a result of bleaching. Several studies reported no evidence of deleterious effects on enamel after applying high concentrations of HP [25, 27]. In contrast, other studies found that high concentrations of HP induced a loss of minerals, thus indicating a demineralizing effect on the enamel during exposure to HP [4, 24]. It was explained that loss of the principal organic component associated with the mineral is related to the detrimental effect on the inorganic content [4, 24], especially during long term applications [28, 29]. However, there is still debate as to whether these agents could adversely affect dental hard tissues in a clinically meaningful manner.

The mechanisms underlying tooth bleaching are still not entirely understood. The mechanism of peroxide-based whitening agents is based specifically on oxidative processes within the hard dental tissues, with active oxygen interacting with the organic components and chromophores. The rate of interaction and the type of active oxygen formed are dependent on the concentration of peroxide as well as on the formulation pH. The strong oxidizing effect of HP on the organic matrix can be increased by a bleaching agent with low pH [30]. Therefore, it might be presumed that studies which reported adverse effects on enamel of bleaching products reflect not the bleaching agent itself but the pH of the formulation [1, 25].

Interventions to overcome the potential deleterious effects of bleaching agents have been proposed, with fluoridated agents being most commonly proposed [2].

The positive effect of highly concentrated fluoride products related to caries prevention and the inhibition of erosion is well described [31, 32]. One mechanism of action for the anticaries effect of topical fluoridation is through formation of a protective fluoride-rich layer consisting of CaF_2_ and CaF_2_-like complexes. Formation of the protective fluoride-containing minerals is favored by high fluoride containing agents with low-pH values. It has been demonstrated that the pH-value plays a very important role in the formation of fluoride-rich complexes [31, 32].

This may explain the finding of the current study that the low pH of the bleaching agent and high concentration of NaF had a synergistic effect on increasing enamel hardness. This effect was observed from the second session of treatment onwards (Figure 1) and the effect was more apparent when NaF was applied after bleaching with the low-pH agent. NaF per se had little effect on increasing enamel hardness (Figure 1). It seems that bleaching with a low-pH product induces some structural alterations [31, 32] of the enamel which improve varying degrees of fluoride uptake to form mineral complexes. However, SEM images did not reveal alteration in surface morphology of enamel after bleaching treatments. Fluoride treatment caused a smooth surface which may be explained by fluoride-containing mineral deposits.

There appeared to be little benefit in combining fluoride treatments with a bleaching agent having a neutral pH (Figure 1). These treatment protocols (G7 and G8) had a delayed effect on increasing enamel surface hardness compared to the specimens treated with acidic bleaching agent (G5 and G6) and their effect was observed after only the third treatment session (Figure 1). In addition, these treatment protocols had no impact on increasing enamel hardness of the unbleached specimens. Nonetheless, fluoride treatments can be useful in the management of dentin hypersensitivity which is often a side effect of tooth bleaching [33, 34].

There were no significant differences in hardness values between post and prebleaching fluoridation. This finding should be cautiously interpreted since this in vitro examination did not reproduce the behavior of the enamel in clinical conditions and since the effect of tooth brushing or chewing and dietary acid exposure was not simulated. Therefore, it might be assumed that the remaining fluoride induced mineral complexes after one application of topical fluoride application, or after bleaching treatment might cause surface hardening of the enamel surface.

This study examined the possible enamel alterations in terms of surface hardness and morphology. Although no morphological alterations resulted from bleaching treatments with high concentration of HP with neutral or acidic pH under in vitro condition. Pola Office led to reduced enamel surface microhardness which was recovered with fluoride therapy. Future studies are needed to test other important variables such as bond strength of conservative [35] and orthodontic [36] materials onto bleached enamel, in order to improve the knowledge about the effect of bleaching products and fluoridation on enamel surface properties.

The main limitation of the present study is that it might not reproduce the behavior of the enamel under the clinical conditions. In addition, the result could be more clearly detected through additional laboratory tests, such as fourier-transform infrared spectroscopy, energy dispersive X-ray analysis, and X-ray diffraction analysis to investigate changes in organic and inorganic components in enamel. Although the hardness test provides no specific information about the structural changes within enamel [37], this test is commonly used to detect mechanical properties of the enamel following bleaching treatment [38]. While the data of one study may not resemble others, the trends of hardness changes can be compared [38, 39]. Additionally, also randomized clinical trials would be needed in the future in order to confirm the results of in vitro studies.

## 5. Conclusions

Within the limitations of this current study, it was concluded that:

1. Pola Office with 35% hydrogen peroxide and pH = 3.85 resulted in a reduction in surface enamel microhardness compared to an unbleached group after three applications. However, bleaching with Pola Office+ with a neutral pH did not result in a significant loss of enamel hardness compared to the unbleached group.
2. Fluoridation before or after bleaching treatment recovered enamel hardness loss for Pola Office group after the second and third sessions of the bleaching treatments.
3. Topical fluoride agent (neutral 2% NaF) after bleaching with Pola Office caused higher enamel hardness compared to the specimens subjected to 2% NaF only at T14.

## Figures and Tables

**Table 1:** Summary of the experimental conditions.

Groups	Treatments
1	Unbleached specimens (negative control)
2	2% neutral sodium fluoride gel^c^ (2% NaF) (positive control)
3	35% Hydrogen peroxide (HP)^a^
4	37.5% HP^b^
5	35% hydrogen peroxide (HP)^a^ +2% NaF at each session for 4 min
6	2% NaF^c^ for 4 min 24 hr before bleaching treatment +35% HP^a^
7	37.5% HP^a^ +2% NaF at each session for 4 min
8	2% NaF for 4 min 24 hr before bleaching treatment +37.5% HP^b^

^a^Pola Office 35% HP (SDI, Bayswater, Victoria, Australia), ^b^Pola Office +37.5% HP (SDI, Bayswater, Victoria, Australia); ^c^Fluor Gel (Maquira Dental Products, Maringá, PR, Brazil).

**Table 2:** In-office bleaching agents used in the study.

Product	Composition
Pola Office liquid	35% Hydrogen peroxide 65% water,
Pola Office powder	Thickener, catalysts, dye, desensitizing agents (potassium nitrate) [14, 15]
Pola Office+	Aqua, hydrogen peroxide, sodium hydroxide, potassium nitrate [16, 17]

**Table 3:** Descriptive values of enamel microhardness in study groups at baseline, 1^th^ (T1), 7^th^ (T7), and 14^th^ (T14) day after bleaching and topical fluoride application (*n* = 10).

Time	Groups	Mean ± SD	Min.	Max.	Median
T0	G1	375.50 ± 23.32	333	412	30
	G2	386.40 ± 23.97	352	416	10
	G3	377.10 ± 19.44	352	400	15
	G4	380.20 ± 21.46	345	406	20
	G5	380.30 ± 24.45	345	413	20
	G6	377.90 ± 15.07	352	406	30
	G7	379.40 ± 20.41	343	406	30
	G8	383.30 ± 21.13	352	411	30

T1	G1	380.30 ± 18.76	406.00	352.00	30
	G2	388.10 ± 18.52	427.00	363.00	10
	G3	364.60 ± 25.69	400.00	317.00	25
	G4	353.30 ± 70.04	434.00	209.00	15
	G5	424.70 ± 52.61	491.00	337.00	25
	G6	361.70 ± 62.92	465.00	308.00	10
	G7	397.00 ± 38.11	457.00	337.00	10
	G8	371.90 ± 50.31	442.00	291.00	20

T7	G1	382.80 ± 21.78	413.00	352.00	10
	G2	383.00 ± 22.55	406.00	347.00	15
	G3	323.80 ± 48.40	381.00	227.00	10
	G4	347.80 ± 38.54	400.00	280.00	10
	G5	418.40 ± 34.98	473.00	352.00	25
	G6	391.90 ± 26.05	427.00	337.00	20
	G7	396.10 ± 52.87	491.00	308.00	20
	G8	401.80 ± 47.14	465.00	308.00	10

T14	G1	390.00 ± 28.46	442.00	347.00	20
	G2	405.80 ± 22.11	434.00	375.00	20
	G3	320.70 ± 35.91	375.00	272.00	20
	G4	332.30 ± 77.04	427.00	245.00	10
	G5	482.30 ± 47.92	548.00	434.00	10
	G6	416.40 ± 56.69	482.00	337.00	10
	G7	420.10 ± 47.97	491.00	352.00	15
	G8	411.40 ± 25.62	449.00	363.00	30

Abbreviations: T0, T1, T7, and T14 are enamel microhardness values at baseline, first, second, and third session of treatment. Negative control specimen (unbleached) (G1); Positive control specimen (2% neutral sodium fluoride) (2% NaF) (G2); specimen bleached with Pola Office (G3); specimen bleached with Pola Office+ (G4); specimen bleached with Pola Office and treated with 2% NaF for 4 min (G5); specimen treated with 2% NaF 24 hr before bleaching process with Pola Office (G6); specimen bleached with Pola Office+ and treated with 2% NaF for 4 min (G7); specimen treated with 2% NaF 24 hr before bleaching process with Pola Office+ (G8).

## Data Availability

Data supporting this research article are available upon request from the corresponding author (fariba.motevaselian@yahoo.com).

## Additional Points

*Clinical Significance*. Due to the low pH of bleaching agents, enamel softening may occur. Neutral sodium fluoride can promote enamel hardness.
